# Neuromuscular performance and biochemical response to maximal anaerobic effort: assessment of longterm adaptations in professional tennis

**DOI:** 10.1038/s41598-026-57914-3

**Published:** 2026-06-22

**Authors:** Tomasz Waldziński, Jan Mieszkowski, Bartłomiej Niespodziński, Paulina Brzezińska, Aleksandra Durzyńska, Magdalena Kochanowicz, Jędrzej Antosiewicz, Andrzej Kochanowicz

**Affiliations:** 1 Faculty of Health Sciences, University of Lomża, Łomża, Poland; 2 Department of Gymnastics and Dance, Gdańsk University of Physical Education and Sport, Gdańsk, Poland; 3 Department of Biological Foundations of Physical Education, Faculty of Health Sciences and Physical Education, Kazimierz Wielki University, Bydgoszcz, Poland; 4 Department of Physical Therapy, Medical University of Gdańsk, Gdańsk, Poland; 5 Department of Bioenergetics and Physiology of Exercise, Medical University of Gdańsk, Gdańsk, Poland

**Keywords:** Wingate anaerobic test, Fatigue, Inflammation, Anaerobic exercises, Neuromuscular adaptations, Health care, Medical research, Physiology

## Abstract

Professional tennis involves repeated short-duration, high-intensity anaerobic efforts interspersed with brief recovery intervals. Powerful serves, explosive sprints, rapid directional changes, and muscular contractions place substantial physiological demands and may lead to distinct long-term adaptations at both neuromuscular and biochemical levels, enabling efficient fatigue management and sustained high-level performance during training and competition. This study aimed to analyse the effect of a long-term professional tennis training adaptations both on neuromuscular and biochemical level in context of the fatigue induced by maximal anaerobic effort (MAnE). 14 professional tennis players (TP) (20.00 ± 0.96 years), and 15 physically active men (PAM) (20.07 ± 1.59 years) finished the study. The testing protocol consisted of MAnE in form of 2 × 30-second Wingate Anaerobic Test and maximal voluntary isometric contractions (MVICs) and submaximal isometric contractions at 20% and 50% of MVIC of the knee extensors and flexors with a surface electromyography evaluation before and after MAnE. Blood samples were collected at baseline, immediately after, and 3 and 24 h after MAnE to assess serum markers, including 8-hydroxy-2′-deoxyguanosine, albumin, interleukins (IL-6, IL-10, IL-15), and total antioxidant capacity (TAC). TP exhibited superior absolute (7.8%, *p* = 0.05) and relative peak power (10.1%, *p* < 0.01) during MAnE in compare with PAM. After the MAnE, TP were able to maintain their performance in form of peak rate of torque development in knee flexion of MVIC, while PAM showed it reduction by 26% (*p* < 0.05). In addition, rectus femoris muscle in TP showed increase in muscle activity (52–62%, *p* < 0.05) after MAnE in both knee flexion and extension at 20% of MVIC. In context of biochemical analysis, TP had higher TAC levels at baseline (47.8%, *p* < 0.01) and 24 h after MAnE (34.7%, *p* < 0.01) compared to PAM. Additionally, while IL-10 concentrations were significantly higher in TP at baseline (22.0%, *p* < 0.05), at 3 h (14.2%, *p* < 0.05) and 24 h after MAnE (26.8%, *p* < 0.05), IL-6 showed attenuated increase after MAnE and its concentrations immediately after (19.8%, *p* < 0.01) and 3 h (27.5%, *p* < 0.01) were lower than PAM. The findings indicate that, compared with physically active men, elite tennis players exhibit distinct neuromuscular and biochemical responses to standardized MAnE, which may reflect long-term training-related physiological adaptations.

## Introduction

Elite tennis is characterized by repeated short-duration, high-intensity actions interspersed with brief recovery intervals. Powerful serves, explosive sprints, accelerations, decelerations, rapid directional changes, and repeated stroke production place substantial demands on anaerobic metabolism, whole-body force production, power transfer, and neuromuscular control^1–4^. These sport-specific demands may drive long-term neuromuscular and biochemical adaptations that support fatigue resistance and sustained high-level performance during training and competition. Accordingly, torque-based indices of maximal force and rapid force production, such as PkTQ and PkRTD, together with SEMG-derived muscle activation, median frequency, and coactivation indices, are relevant for characterizing fatigue-related neuromuscular performance under standardized maximal-effort conditions^2,3,5,6^. In parallel, repeated high-intensity intermittent efforts may challenge redox homeostasis and modulate inflammatory signaling, which supports the assessment of total antioxidant capacity (TAC), 8-hydroxy-2′-deoxyguanosine (8-OHdG), and cytokines such as interleukin-6 and -10 (IL-6 and IL-10) as complementary markers of post-exercise stress and recovery^7–10^.

Repeated maximal anaerobic efforts (MAnE) can impair performance through peripheral fatigue and central limitations, which are particularly relevant in intermittent sports such as tennis^11^. Neuromuscular fatigue induced by anaerobic exercise alters muscle activation, force-generating capacity, and intermuscular coordination^5^. Trained athletes often show adaptations that preserve rapid force production and enhance fatigue resistance. Proposed mechanisms include enhanced neural drive, more efficient motor unit recruitment, and reduced antagonist coactivation, all of which can improve performance during high-intensity efforts^12,13^. Such adaptations are particularly relevant in tennis, where repeated high-intensity actions must be maintained across prolonged intermittent play.

Beyond neuromuscular factors, exercise-induced inflammation and oxidative stress contribute to fatigue and recovery during repeated anaerobic exercise. Acute exercise is commonly accompanied by transient elevations in circulating cytokines, particularly pro-inflammatory mediators^7^. This inflammatory milieu is considered one of the key factors that can constrain performance and delay post-exercise recovery^14^. Recent studies have emphasized the role of cytokine dynamics after intense anaerobic exercise, including marked increases in IL-6 together with a compensatory rise in IL-10^7,15,16^. Waldziński et al.^17^ demonstrated that MAnE elicits distinct cytokine profiles that may reflect training-related adaptations, including differences in both pro- and anti-inflammatory mediators compared with physically active controls. Importantly, acute inflammatory responses can vary by sport discipline and by the specific pattern of muscle work. In line with this, Kochanowicz et al.^18^ reported differential cytokine responses to upper-body versus lower-body Wingate Anaerobic Tests (WAnTs) in elite artistic gymnasts, supporting the view that long-term training modulates metabolic and inflammatory pathways.

In parallel, anaerobic exercise challenges redox homeostasis. Oxidative DNA damage (8-OHdG) and TAC provide complementary information on oxidative stress and antioxidant defense following intense exercise^8,9^. Together with inflammatory cytokines, these indices enable a more comprehensive evaluation of the biological processes underpinning fatigue, recovery, and sport-specific adaptation.

Despite these advances, the integrated assessment of neuromuscular and biochemical responses to MAnE in professional tennis players remains limited. Most studies have examined neuromuscular or biochemical fatigue in isolation rather than addressing their combined responses^19–23^. Because fatigue and recovery are shaped by interacting neuromuscular and biochemical processes, simultaneous assessment is important. However, evidence on how long-term tennis specialization modulates this interplay remains scarce.

Thus, the objective of the present study was to evaluate neuromuscular performance and acute post-exercise biochemical responses in professional tennis players versus physically active controls following repeated MAnE.

## Materials and methods

### Experimental overview

This study was a controlled, cross-sectional quasi-experimental design aimed at comparing neuromuscular performance and biochemical responses to MAnE between two groups: professional tennis players (TP) and physically active men (PAM). All assessments were performed over a two-day protocol, with main testing procedures conducted on Day 1 and delayed biochemical sampling completed on Day 2.

Two weeks prior to testing, all participants completed a familiarization session to minimize learning effects and ensure consistency in performance, particularly during the MAnE. On the main testing day, participants reported to the laboratory in the morning (between 8:00 and 11:00 a.m.). The testing protocol began with fasting blood sampling. Immediately afterward, body composition was assessed using anthropometric measurements and bioelectrical impedance analysis (InBody 720, South Korea, Seoul). Participants then performed a standardized warm-up consisting of 5 min of submaximal cycling on a mechanically braked ergometer (Monark 894E, Peak Bike, Sweden) according with the Bar-Or^24^ and Kochanowicz et al.^25^ modifications. This was followed by neuromuscular performance with simultaneous surface electromyography (SEMG) assessments before and after MAnEs. Blood samples were again collected after the MAnE. Finally, 24 h post-exercise, participants had last blood sampling before which they were instructed to abstain from any physical activity and to maintain their habitual diet without deviations in caloric intake or meal timing.

## Participants

Twenty-nine young adult males aged 18–22 years volunteered to participate in this study. The experimental group consisted of 14 professional TP, while the control group included 15 PAM. All participants reported good general health and were free from neuromuscular, inflammatory, or metabolic disorders. The characteristics of each group are shown in Table 1.

**Table 1:** Physical characteristics in tennis players and physically active men (mean ± standard deviation).

Variable	Unit	Physically active men (*n* = 15)	Tennis players (*n* = 14)	t	*p* value
Age	years	20.00 ± 0.96	20.07 ± 1.59	-0.31	0.89
Body height	cm	184.78 ± 6.49	184.96 ± 5.08	0.04	0.93
Body mass	kg	80.84 ± 8.72	77.28 ± 5.68	1.29	0.21
BMI	kg · m^−2^	23.81 ± 2.37	22.77 ± 1.02	1.74	0.14
Percent body fat	%	13.62 ± 5.99	7.53 ± 2.32	3.54	< 0.01*

* significant difference at *p* < 0.01.

The TP were classified as elite athletes and actively competed in both national-level tournaments and international ITF events. At the time of recruitment, all players were ranked within the top 40 of Polish National Men’s Tennis Association ranking. They were recruited from regional high-performance training centers and had a minimum of eight years of continuous training experience. Their weekly regimen consisted of at least five structured training sessions, with each session lasting approximately 2–3 h and encompassing technical, tactical, and physical conditioning components. Thus, their weekly tennis-specific training exposure was approximately 10–15 h during the off-season transition period.

The control group consisted of male physical education students who did not participate in competitive sports. They reported engaging in no more than five hours per week of moderate-intensity physical activity, which was primarily carried out through mandatory curricular classes including soccer, volleyball, gymnastics, combat sports, and swimming.

Participants were excluded if they had: (1) taken any drugs or dietary supplements, ergogenic aids, or sports nutrition products, including creatine and caffeine-containing products, in the four weeks preceding the study; (2) sustained musculoskeletal injuries in the three months prior to data collection; (3) participated in structured anaerobic or resistance training programs during the previous year (applicable to PAM only). The study was conducted at the end of October, during the off-season transition period after the tournament season. During this period, none of the participants reported using dietary supplements, ergogenic aids, or sports nutrition products. All participants were instructed to refrain from any physical activity, caffeine, and alcohol for at least 48 h prior to testing.

Written informed consent was obtained from all subjects following a detailed explanation of the procedures. The study protocol was approved by the Bioethics Committee of the University of Physical Education and Sport in Gdańsk (Resolution No. 2, dated December 16, 2024), and all procedures conformed to the Declaration of Helsinki.

### Maximal anaerobic effort

The WAnT was used as a standardized lower-body maximal anaerobic exercise stimulus to induce acute fatigue and enable between-group comparison of neuromuscular and biochemical responses. It was not intended to replicate the technical or whole-body movement demands of tennis match play.

The MAnE was performed using 30-second WAnT conducted on a mechanically braked bicycle ergometer (Monark Ergomedic 894E, Vansbro, Sweden) which was repeated again after 30-second passive recovery period. All test performance was made according with the Bar-Or and modification proposed by Kochanowicz et al.^24,25^. Each WAnT was preceded by a 3-second unloaded acceleration phase, after which the preset braking resistance was applied manually. The load was set at 7.5% of the participant’s body mass. The bicycle ergometer was linked to a personal computer that operated the MCE 5.1 software^26^ which allowed to calculate: peak power output, mean power output of each WAnT. All performance parameters were expressed in both absolute values (W) and relative values normalized to body mass (W · kg^−1^). The decline in power output between the first and second WAnT was used to evaluate short-term recovery capacity. Throughout the whole testing phases, verbal encouragement was provided from start to finish to help maintain the highest possible cadence during testing.

### Neuromuscular performance

Neuromuscular performance of the dominant lower limb was assessed before and 3 min after the MAnE, in form of isometric strength testing synchronized with SEMG. Dominant limb was identified based on participant leg preferentially used to kick a ball^27,28^.

All neuromuscular measurements were conducted in a controlled laboratory environment with participants seated on an isokinetic dynamometer (Biodex System 4, Biodex Medical Systems Inc., Shirley, NY, USA). The hip and knee joints were fixed at 90° flexion, and the distal shin of the dominant leg was attached to the dynamometer lever arm using a padded strap to minimize extraneous movement and ensure consistent torque transmission. All measurements were conducted in accordance with the manufacturer’s operating guidelines to ensure standardized positioning, calibration, and force acquisition protocols. The reliability of the Biodex System 4 dynamometer has been extensively validated in previous research, demonstrating good to excellent test–retest reproducibility depending on the joint complex and functional parameter assessed^29–31^.

Participants first performed three maximal voluntary isometric contractions (MVICs) of the knee extensors and flexors, each lasting 3 s, with 60 s of rest between trials. Participants were asked to extend or flex their knee as hard and as fast as possible. Strong verbal encouragement and visual feedback were provided. Five minute after completing the MVICs, participants performed submaximal isometric contractions at 20% and 50% of MVIC. These were performed for both knee extension and flexion, with visual feedback provided on screen showing real-time torque and the target value. The order of target levels (20% or 50% MVIC) as well as function (extension or flexion) were randomized. For each intensity, participants were instructed to reach and hold the target torque for 3 s. Each submaximal contraction was repeated three times, separated by 30 s of rest.

The similar procedure was performed 3 min after the MAnE, with single repetition of each part of testing and 30 s between each activity. This approach was chosen to reduce the time for recovery after the MAnE.

SEMG was recorded using a wireless electromyograph TeleMyo DTS (Noraxon Inc., Scottsdale, AZ, USA). Bipolar Ag/AgCl electrodes (1 cm^2^ of active area; Sorimex, Toruń, Poland) were placed on rectus femoris, vastus lateralis, vastus medialis, in accordance with SENIAM recommendations^32^. Interelectrode distance was 2 cm; skin was shaved, abraded, and cleaned with alcohol. SEMG signals were sampled at 1500 Hz, band-pass filtered (10–500 Hz), full-wave rectified, and smoothed using a root-mean-square (RMS) algorithm with a 100 ms moving window. Due to inherent SEMG signal variability, the mean RMS amplitude across the all trials was used for analysis. Submaximal RMS amplitudes (from 20% to 50% MVIC trials) were normalized (NRMS) to the mean RMS value recorded during a one-second time frame taken from the middle of the MVIC. All SEMG analyses were performed using MyoResearch software (ver. 1.08; Noraxon Inc., Scottsdale, AZ, USA).

For neuromuscular performance, following outcome variables were analysed: peak torque (PkTQ, Nm) and peak rate of torque development (PkRTD, Nm · s^−1^) as highest slope of the torque-time curve during the MVIC; muscle activation as mean NRMS (%) and coactivation index (CI, %) during submaximal isometric contractions, mean amplitude (µV), median frequency of power spectrum (Hz) and CI during MVIC.

CI was expressed according to equation^33^:

$${\text{CI }} = \frac{{mean~RMS~of~the~muscle~acting~as~an~antagonist}}{{mean\;RMS~of~the\;muscle~acting~as~an\;agonist~during\;MVIC}}$$

The PkTQ and PkRTD were analysed in absolute values, but also normalized to body mass (Nm · kg^−1^) and PkTQ (Nm · s^−1^ · Nm^−1^), respectively.

All neuromuscular testing procedures were conducted by the same trained investigator to minimize inter-rater variability.

### Biochemical analyses

Venous blood samples (5 mL) were collected from the antecubital vein at four time points: before exercise (fasted state), immediately after MAnE, 3 h, and 24 h post-exercise. Blood was drawn using BD Vacutainer Clot Activator Tubes (Becton Dickinson and Company, Franklin Lakes, NJ, USA) by a qualified medical diagnostican.

After collection, the samples were allowed to clot at room temperature (45 min) and centrifuged at 4000 × g for 10 min in room temperature. In the next stage obtained serum was aliquoted into 500 µL Eppendorf tubes and stored at − 80 °C for no longer than 6 months. All biochemical analyses were performed in a single batch in repetitions after the completion of the experimental protocol.

The following serum biomarkers were assessed: oxidative DNA damage marker: 8-hydroxy-2′-deoxyguanosine (8-OHdG); Inflammatory cytokines: Interleukin-6 (IL-6), Interleukin-10 (IL-10), Interleukin-15 (IL-15); total antioxidant capacity (TAC); serum albumin (ALB).

Concentrations of IL-6, IL-10, IL-15, and ALB were quantified using commercially available high-sensitivity ELISA kits (DRG International Inc., Springfield, NJ, USA) and read using a Thermo Fisher Scientific ELISA Analyser (Thermo Fisher Scientific, Waltham, MA, USA). TAC levels were determined using the TAC Fast Track ELISA kit (LDN-DM P-4100) from Omnignostica Forschungs GmbH (Höflein, Austria). The 8-OHdG concentration was measured using the Human 8-OHdG ELISA kit (Shanghai SunRed Biological Technology Co. Ltd., Shanghai, China). All procedures followed the manufacturers’ instructions, and internal quality controls were implemented to ensure assay precision. Absorbance values were measured in duplicate, and intra-assay coefficients of variation (CV) below 10% were considered acceptable.

### Statistical analysis

All statistical procedures were performed using Statistica software (version 12.2, StatSoft Inc., Tulsa, OK, USA). Descriptive statistics are reported as mean ± standard deviation (SD). Prior to parametric testing, the Shapiro–Wilk test was used to assess normality, and Levene’s test was used to verify the homogeneity of variance. Differences in physical characteristics and changes in SEMG variables (during MVIC) between TP and PAM were analysed using independent-samples t-tests.

The differentiating effects of group (TP vs. PAM), time and their interaction were examined using two-way ANOVAs with repeated-measures. Specifically, first set of ANOVAs was used to compare performance (peak power, mean power, fatigue index) between the first and second WAnT trials during MAnE evaluation.

The second set of ANOVAs was used to compare outcome of neuromuscular performance (PkTQ, PkRTD and SEMG variables during submaximal contractions) pre and post MAnE. Third set of ANOVAs was used to compare outcome of biochemical markers (IL-6, IL-10, IL-15, ALB, TAC, and 8-OHdG): before, immediately after, 3 h post and 24 post the MAnE.

In the case of statistically significant main effects or group × time interactions, Tukey’s post-hoc tests were conducted to determine pairwise differences.

To control for multiplicity arising from testing multiple outcomes, a Bonferroni correction was applied to the p-values of the primary ANOVA main effects (group and repeated-measures factor) and the group × repeated-measures interaction terms.

Effect sizes were calculated using Cohen’s d-value and partial eta-squared (η^2^) statistics and interpreted as follows: d ≥ 0.2 or η^2^ ≥ 0.01—small effect, d ≥ 0.5 or η^2^ ≥ 0.06—moderate effect, d ≥ 0.8 or η^2^ ≥ 0.14—large effect^34^. The level of statistical significance was set at *p* < 0.05.

Additionally, sample size estimations were conducted a priori using G*Power software (version 3.1.9.4; Universität Kiel, Germany). A minimum of 12 participants (for ANOVA 2 × 4) and 16 participants (for ANOVA 2 × 2) were determined to be sufficient to detect large effects with a statistical power of 0.80 and α = 0.05.

## Results

To determine whether long-term tennis training was associated with differences in anaerobic power output and short-term performance maintenance, MAnE outcomes were compared between TP and PAM across two repeated 30-s WAnTs. The results of MAnE are presented in Table 2. Regardless of the repeated-measures factor (1st WAnT vs. 2nd WAnT), TP demonstrated higher performance compared to the PAM in both absolute (7.8%, *p* = 0.05) and relative (10.1%, *p* < 0.01) peak power, as well as in relative mean power (8.8%, *p* < 0.01). A significant effect of repeated measures was observed, with marked declines in performance during the 2nd WAnT, where absolute peak power decreased by 28.9% (*p* < 0.01), absolute mean power by 34.1% (*p* < 0.01), relative peak power by 29.1% (*p* < 0.01), and relative mean power by 34.0% (*p* < 0.01), regardless of the group. No interaction between factors was observed in all MAnE performance variables.

**Table 2:** Maximal anaerobic effort results in tennis players and physically active men.

Variable	Unit	Physically active men (*n* = 15)		Tennis players (*n* = 14)			ANOVA		
		1st WAnT	2nd WAnT	1st WAnT	2nd WAnT		G	RM	G×RM
		Mean ± SD	Mean ± SD	Mean ± SD	Mean ± SD				
Absolute peak power	W	769.98 ± 99.03	536.78 ± 62.62	813.25 ± 81.05	588.08 ± 67.55	p	0.05	< 0.01*	0.80
						η^2^	0,13	0.13	0.01
Absolute mean power	W	607.85 ± 56.27	405.00 ± 52.27	648.68 ± 62.94	422.82 ± 84.12	p	0.17	< 0.01*	0.32
						η^2^	0.07	0.92	0.04
Relative peak power	W · kg^**−1**^	9.62 ± 1.14	6.71 ± 0.73	10.55 ± 0.97	7.61 ± 0.64	p	0.01*	< 0.01*	0.94
						η^2^	0.34	0.85	0.01
Relative mean power	W · kg^**−1**^	7.59 ± 0.44	5.05 ± 0.52	8.41 ± 0.61	5.46 ± 0.95	p	0.01*	< 0.01*	0.18
						η^2^	0.27	0.92	0.06

WAnT, Wingate Anaerobic Test; G, group factor; RM, repeated measure factor;

*significant difference at *p* < 0.01.

**Fig. 1:**
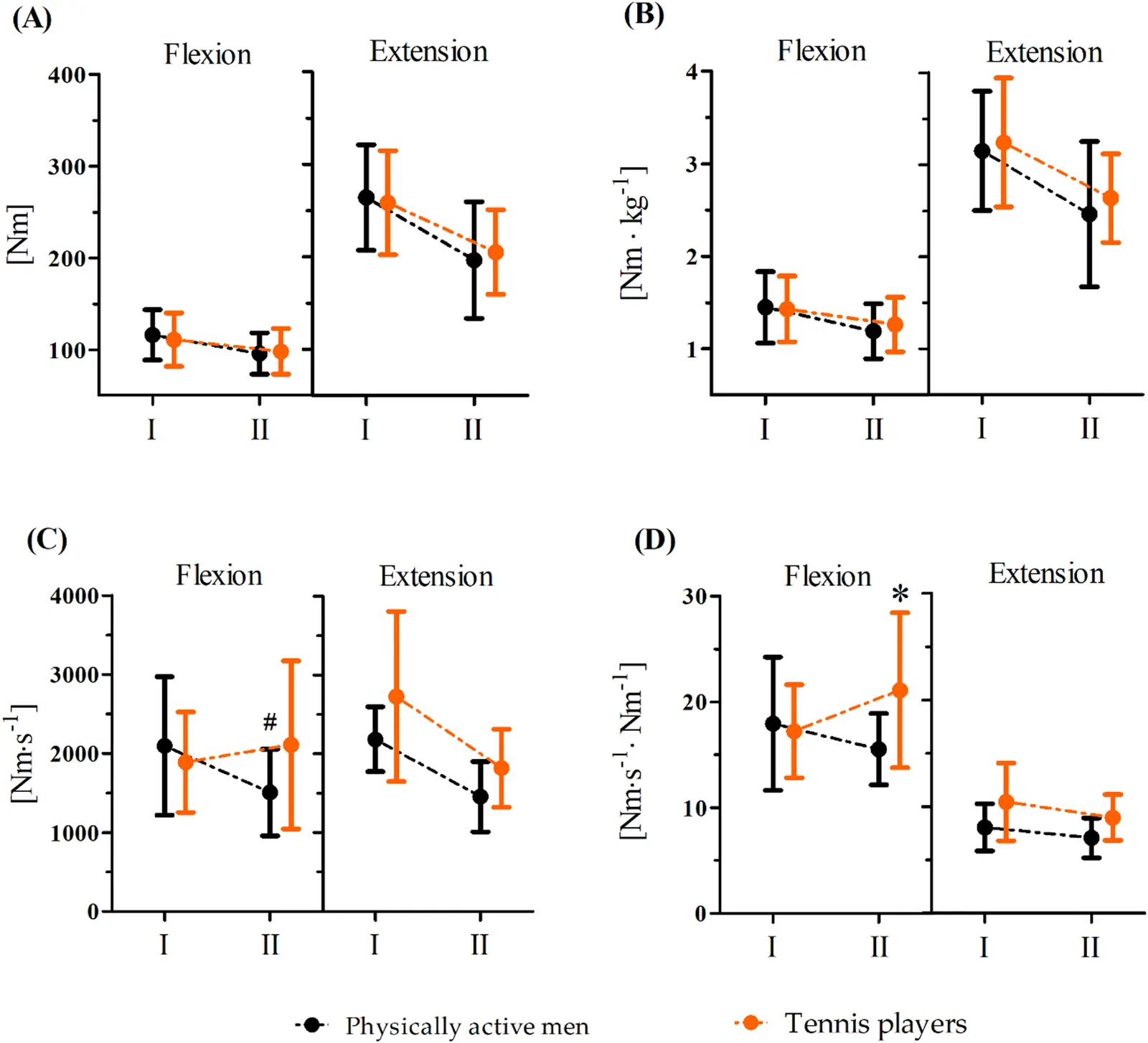
Changes of knee muscle strength in tennis players (*n* = 14) and physically active men (*n* = 15). Absolute (**A**) and normalized (**B**) peak torque; absolute (**C**) and normalized (**D**) peak rate of torque development during knee flexion and extension pre (I) and post (II) maximal anaerobic effort. Dots and whiskers represent mean and standard deviation, respectively. Statistical analysis: *significant difference with physically active men at particular time point at *p* < 0.05; ^#^significant difference with I time point in physically active men at *p* < 0.05.

To assess whether MAnE induced different changes in maximal and explosive muscle strength between groups, PkTQ and PkRTD were analysed before and after the exercise protocol. The neuromuscular performance outcomes are presented in Fig. 1. Two-way ANOVA with repeated-measures revealed a significant main effect of time (pre- vs. post-MAnE) on PkTQ in both knee flexion (*p* < 0.01, η^2^ = 0.54) and extension (*p* < 0.01, η^2^ = 0.69), as well as on normalized PkTQ in knee flexion (*p* < 0.01, η^2^ = 0.53) and extension (*p* < 0.01, η^2^ = 0.68). Regardless of the group factor, PkTQ decreased by 15.0% in knee flexion and by 23.3% in knee extension after the MAnE, while normalized PkTQ decreased by 14.7% and 23.0%, respectively. A significant main effect of time was also observed for PkRTD in knee extension (*p* < 0.01, η^2^ = 0.40), which decreased by 33.2% after the MAnE (*p* < 0.01). Nevertheless, regardless of time factor, both absolute PkRTD and normalized PkRTD were significantly higher in TP during extension compared to PAM, by 19.8% (*p* < 0.05, η^2^ = 0.16) and 18.5% (*p* < 0.05, η^2^ = 0.20), respectively. A significant group-by-time interaction was found for both absolute (*p* < 0.05, η^2^ = 0.15) and normalized (*p* < 0.05, η^2^ = 0.16) PkRTD in knee flexion. Post hoc comparisons revealed a significant reduction of 26.8% (*p* < 0.05) in absolute PkRTD in PAM (Fig. 1C). Comparable outcome was observed for normalized PkRTD in knee flexion (Fig. 1D), with post-MAnE values significantly lower (by 26.2%, *p* < 0.05) in PAM compared to TP.

To examine whether MAnE altered maximal contraction-related SEMG characteristics differently between groups, changes in amplitude and median frequency during MVIC were compared between TP and PAM. These results are shown in Table 3.

**Table 3:** Changes of the amplitude and median frequency of power spectrum in maximal voluntary isometric contraction after maximal anaerobic effort.

Muscle	Knee function	Variable	Group	Baseline value	Change [%]			
				Mean ± SD	Mean ± SD	t	p	d
Rectus femoris	Extension	Mean amplitude [µV]	TP	449.92 ± 129.39	0.21 ± 27.61	− 0.07	0.93	0.02
			PAM	496.14 ± 213.77	0.80 ± 42.54			
		Median frequency [Hz]	TP	75.29 ± 16.03	− 12.88 ± 17.74	0.13	0.89	0.00
			PAM	75.36 ± 16.04	− 12.88 ± 10.28			
	Flexion	Coactivation [%]	TP	5.49 ± 2.80	36.74 ± 79.71	− 1.22	0.23	− 0.45
			PAM	7.23 ± 3.10	8.59 ± 37.83			
		Median frequency [Hz]	TP	43.67 ± 14.51	8.17 ± 34.94	− 0.51	0.60	− 0.20
			PAM	50.98 ± 11.20	1.35 ± 32.08			
Vastus lateralis	Extension	Mean amplitude [µV]	TP	578.35 ± 299.55	− 20.20 ± 27.53	1.83	0.08	0.73
			PAM	475.25 ± 207.03	− 2.65 ± 19.62			
		Median frequency [Hz]	TP	57.21 ± 9.41	8.83 ± 12.21	− 0.37	0.71	− 1.39
			PAM	58.26 ± 11.80	− 10.92 ± 15.94			
	Flexion	Coactivation [%]	TP	4.31 ± 2.48	30.35 ± 108.93	− 1.43	0.16	− 0.58
			PAM	7.83 ± 8.64	− 17.8 ± 41.66			
		Median frequency [Hz]	TP	58.33 ± 9.46	− 6.09 ± 21.67	0.05	0.95	0.02
			PAM	59.25 ± 20.49	− 5.55 ± 22.94			
Vastus medialis	Extension	Mean amplitude [µV]	TP	552.66 ± 384.68	2.60 ± 23.33	− 1.38	0.17	0.51
			PAM	475.14 ± 157.16	26.20 ± 61.60			
		Median frequency [Hz]	TP	61.06 ± 10.75	− 5.14 ± 20.52	− 0.84	0.41	− 0.32
			PAM	60.71 ± 13.16	− 10.43 ± 11.91			
	Flexion	Coactivation [%]	TP	10.95 ± 10.47	0.71 ± 23.58	0.34	0.75	0.13
			PAM	7.62 ± 3.89	7.05 ± 65.21			
		Median frequency [Hz]	TP	49.13 ± 6.93	− 4.28 ± 13.19	1.66	0.10	0.65
			PAM	46.47 ± 7.92	3.18 ± 9.59			

*TP* tennis players,* PAM* physically active men.

**Fig. 2:**
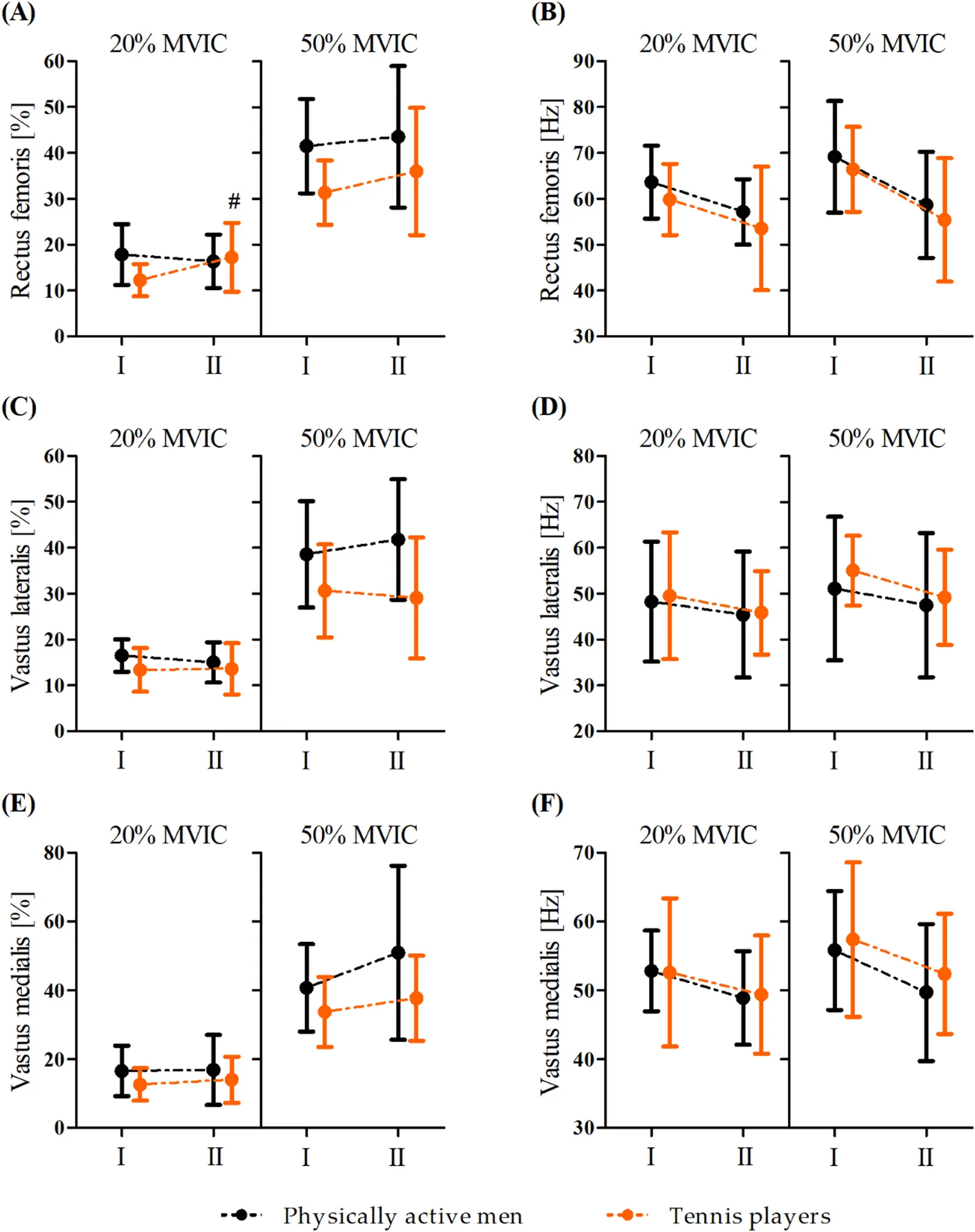
Changes in surface electromyographic activity of knee joint agonist muscles in tennis players (*n* = 14) and physically active men (*n* = 15). Muscle activation (**A**, **C**, **E**) and median frequency of the signal power spectrum (**B**, **D**, **F**) during submaximal knee extensions pre (I) and post (II) maximal anaerobic effort. MVIC—maximal voluntary isometric contraction. Dots and whiskers represent mean and standard deviation, respectively. Statistical analysis: ^#^ significant difference with I time point at *p* < 0.05.

To further characterize fatigue-related neuromuscular responses, agonist muscle activation and SEMG median frequency during submaximal knee extension were analysed at 20% and 50% of MVIC. These results are shown in Fig. 2. ANOVA revealed significant main effects of group (TP vs. PAM) for muscle activation in the rectus femoris (*p* < 0.05, η^2^ = 0.14) and vastus lateralis (*p* < 0.05, η^2^ = 0.22) at 50% MVIC. Regardless of the time factor, muscle activation in TP was 21.4% lower for the rectus femoris and 27.5% lower for the vastus lateralis compared with PAM. A significant main effect of time was also observed for the vastus medialis at 50% MVIC (*p* < 0.05, η^2^ = 0.16), indicating an 18.7% increase following MAnE independent of group. In addition, at 20% MVIC, a significant group × time interaction for rectus femoris activation (*p* < 0.05, η^2^ = 0.22) demonstrated a post-MAnE increase specific to TP, with rectus femoris activation rising by 62.1% (*p* < 0.05). For SEMG median frequency, significant main effects of time were detected for the rectus femoris at both 20% MVIC (*p* < 0.01, η^2^ = 0.26) and 50% MVIC (*p* < 0.01, η^2^ = 0.39), for the vastus lateralis at 50% MVIC (*p* < 0.05, η^2^ = 0.13), and for the vastus medialis at 20% MVIC (*p* < 0.01, η^2^ = 0.24) and 50% MVIC (*p* < 0.05, η^2^ = 0.20). Regardless of group factor, median frequency decreased following the WAnTs in the rectus femoris by 10.0% at 20% MVIC and by 15.7% at 50% MVIC; in the vastus lateralis by 8.7% at 50% MVIC; and in the vastus medialis by 6.7% at 20% MVIC and by 10.1% at 50% MVIC.

**Fig. 3:**
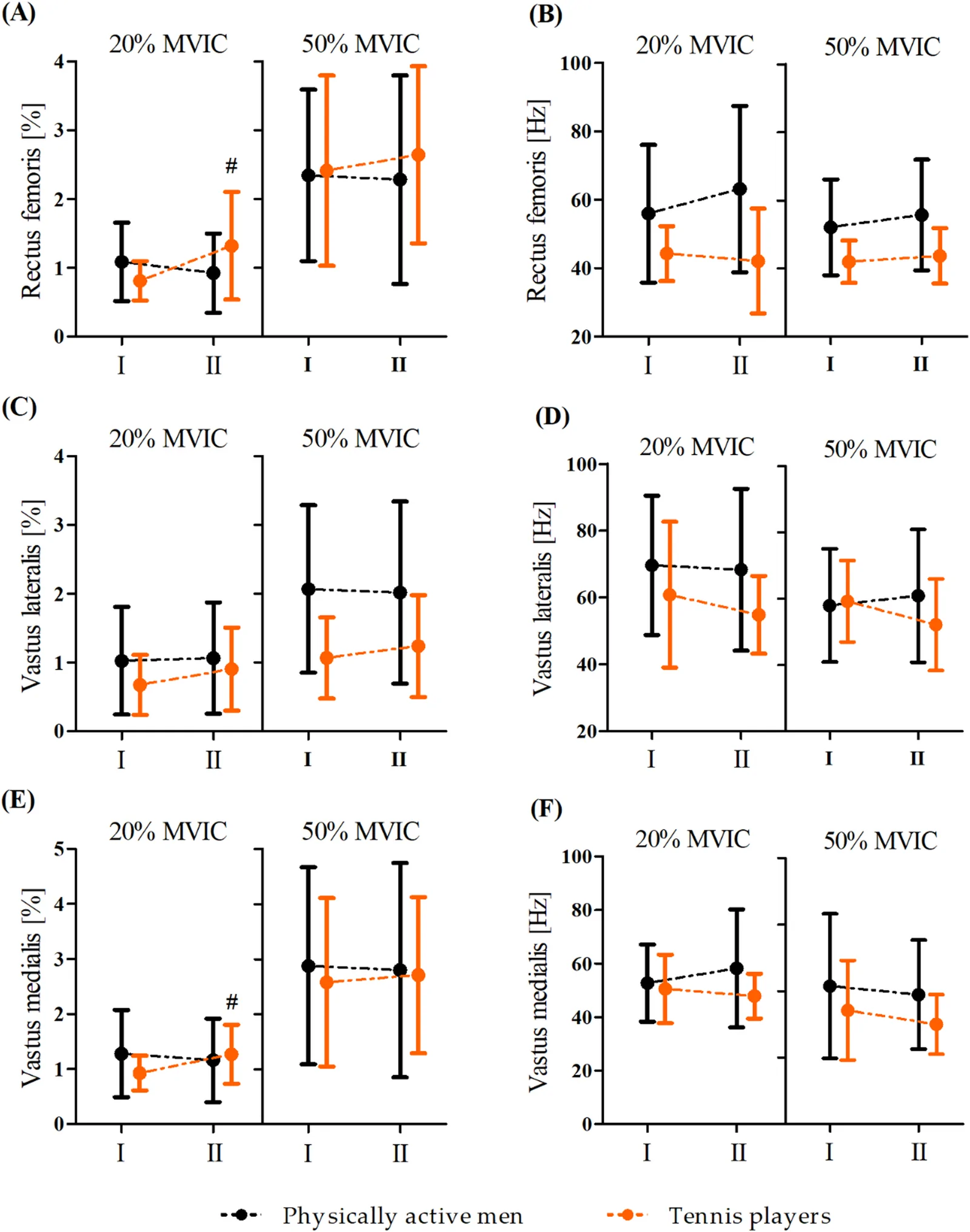
Changes in surface electromyographic activity of knee joint antagonist muscles in tennis players (*n* = 14) and physically active men (*n* = 15). Coactivation index (**A**, **C**, **E**) and median frequency of the surface electromyography signal power spectrum of antagonistic muscle (**B**, **D**, **F**) during submaximal knee flexions pre (I) and post (II) maximal anaerobic effort. Dots and whiskers represent mean and standard deviation, respectively. MVIC- maximal voluntary isometric contraction. Statistical analysis: ^#^significant difference with I time point at *p* < 0.05.

To evaluate antagonist muscle involvement after MAnE, CI values and SEMG median frequency of the quadriceps muscles were analysed during submaximal knee flexion. These results are shown in Fig. 3. A significant main effect of group (TP vs. PAM) was found for the vastus lateralis at 50% MVIC (*p* < 0.05, η^2^ = 0.13), with CI in TP being 53.8% lower than in PAM. At 20% MVIC, significant group × time interactions were observed for the rectus femoris (*p* < 0.05, η^2^ = 0.17) and vastus medialis (*p* < 0.05, η^2^ = 0.20). Post hoc comparisons indicated that, following MAnE, CI increased in TP by 52.1% for the rectus femoris (*p* < 0.05) and by 20.1% for the vastus medialis (*p* < 0.05). For the median frequency, a significant main effect of group was detected only for the rectus femoris during knee flexion at both 20% MVIC (*p* < 0.01, η^2^ = 0.26) and 50% MVIC (*p* < 0.01, η^2^ = 0.39). Across both time points, median frequency values were lower in TP than in PAM, by 27.1% at 20% MVIC and by 20.8% at 50% MVIC.

**Fig. 4:**
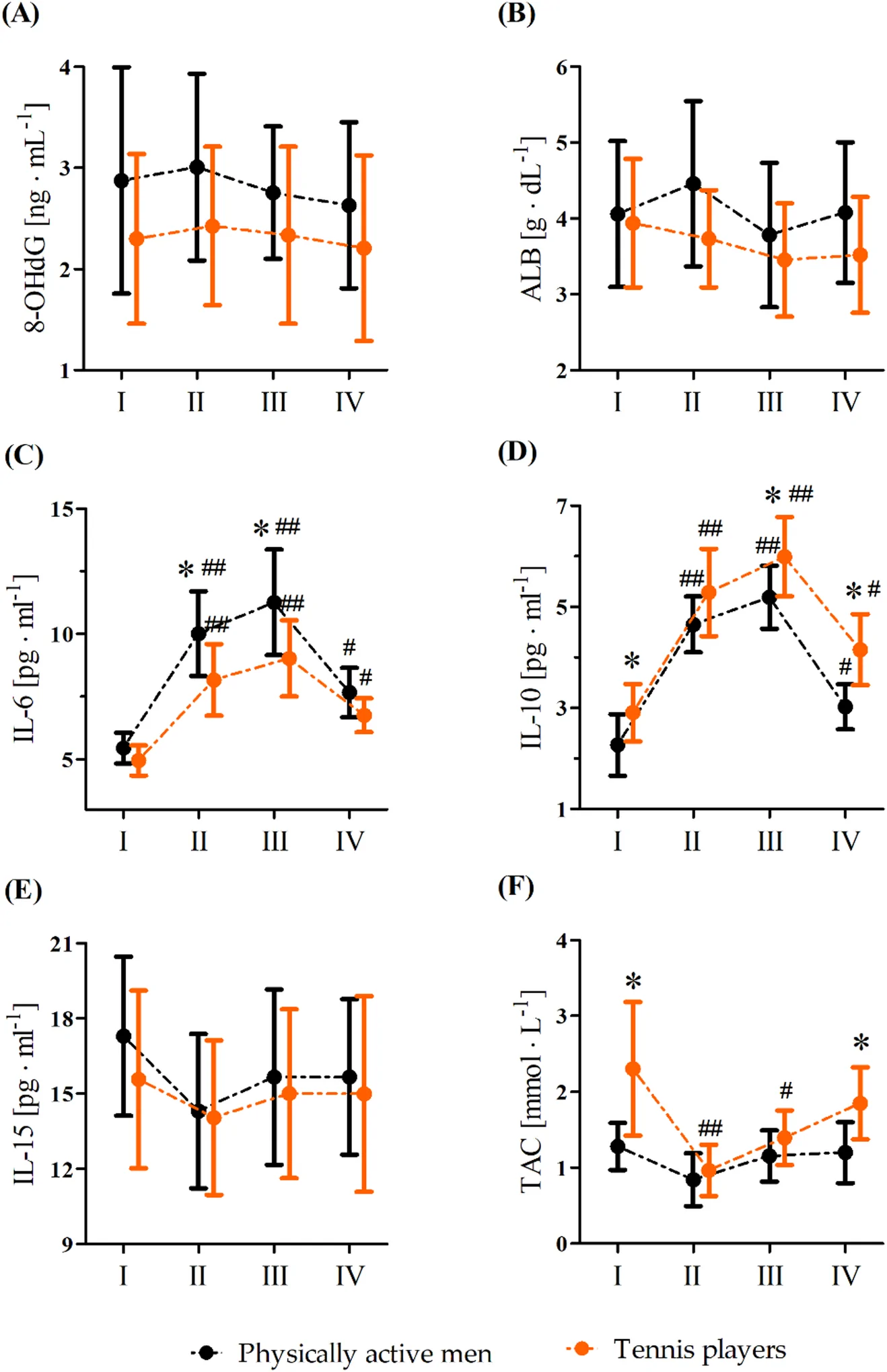
Changes in serum concentrations of selected markers of oxidative stress, inflammation and muscle damage induced by maximal anaerobic effort (MAnE) in tennis players (*n* = 14) and physically active men (*n* = 15). 8-hydroxy-2′-deoxyguanosine (**A**); albumin (**B**); interleukin 6 (**C**); interleukin 10 (**D**); interleukin 15 (**E**); total antioxidant capacity (**F**); pre- (I), post-0 (II), post-3 h (III) and post-24 h (IV) after MAnE; Dots and whiskers represent mean and standard deviation, respectively. Statistical analysis: *significant difference between tennis players and physically active men at particular time point at *p* < 0.01; ^#^significant difference vs. I in particular group at *p* < 0.01, ^##^significant difference vs. I and IV in particular group at *p* < 0.01.

To determine whether MAnE induced different inflammatory and redox-related responses between groups, serum cytokines, TAC, 8-OHdG, and ALB were analysed before and after the exercise protocol. These results are presented in Fig. 4. Analysis of variance revealed a significant main effect of time for IL-6 (*p* < 0.01, η^2^ = 0.91), IL-10 (*p* < 0.01, η^2^ = 0.94), IL-15 (*p* < 0.01, η^2^ = 0.14), and TAC (*p* < 0.01, η^2^ = 0.43). Regardless of group, IL-6 increased by 75.0% immediately after MAnE (*p* < 0.01), and IL-10 increased by 76.9% (*p* < 0.01), with both cytokines remaining elevated and reaching their highest concentrations 3 h post-exercise. In contrast, IL-15 decreased by 13.5% relative to baseline (*p* < 0.01), and TAC decreased by 47.5% (*p* < 0.01) following MAnE. For TAC, the group × time interaction was significant (*p* < 0.01, η^2^ = 0.17), indicating a group-specific post-exercise response. Specifically, only TP exhibited a significant immediate reduction in TAC, amounting to a 13.5% decrease from baseline (*p* < 0.01). In addition, TAC was consistently higher in TP than in PAM, with baseline values higher by 47.8% (*p* < 0.01) and values at 24 h post-MAnE higher by 34.7% (*p* < 0.01). For IL-10, a significant main effect of group (*p* < 0.01, η^2^ = 0.41), together with a significant group × time interaction (*p* < 0.01, η^2^ = 0.17), demonstrated higher IL-10 concentrations in TP compared with PAM at multiple time points. TP exceeded PAM by 22.0% at baseline (*p* < 0.05), by 14.2% at 3 h post-MAnE (*p* < 0.05), and by 26.8% at 24 h post-MAnE (*p* < 0.05). IL-6 also displayed a significant main effect of group (*p* < 0.01, η^2^ = 0.45), with overall IL-6 concentrations being 15.9% lower in TP than in PAM. This was further supported by a significant group × time interaction (*p* < 0.01, η^2^ = 0.25), showing an attenuated post-exercise rise in TP. Accordingly, IL-6 concentrations in TP were 19.8% lower immediately after MAnE (*p* < 0.01) and 27.5% lower at 3 h post-MAnE (*p* < 0.01) relative to PAM. In contrast, neither 8-OHdG nor ALB showed significant main effects of time, group, or their interaction. Both markers, however, exhibited non-significant trends, with 8-OHdG tending to increase transiently after exercise and ALB to decrease, while mean concentrations of both were consistently lower in TP compared with PAM across all time points.

## Discussion

To our knowledge, this is the first study to comprehensively examine both neuromuscular and biochemical responses to MAnE in elite TP, directly compared with PAM. By integrating torque-based performance measures, muscle activation, and key inflammatory and oxidative stress biomarkers, this investigation provides a multidimensional perspective on fatigue resistance and recovery potential under standardized maximal anaerobic effort conditions in elite male tennis players with long-term high-performance training experience.

Considering the neuromuscular response to the MAnE, the primary finding was a reduction in knee flexion PkRTD in the PAM group, whereas the TP group preserved their capacity for explosive torque production. Concurrently, knee extension PkRTD was higher in TP than in PAM. Notably, the post-MAnE reduction in PkRTD was evident for both absolute and normalized values. By comparison, PkTQ decreased after MAnE to a similar extent in both groups for both knee flexion and knee extension. Thus, suggest that TP fatigue resistance was associated in factors different than capability to exert maximal muscle strength. It is known, that the maximal, short all-out anaerobic efforts substantially impair force-generating capacity due to peripheral fatigue mechanisms such as metabolite accumulation, impaired excitation–contraction coupling and reduced muscle contractile capacity^35,36^. However, considering RTD, the intrinsic muscle factors play a minor role compared with the neural factors^6^. Therefore, the attenuated loss of the performance of knee flexors in TP should be related to long-term tennis-specific adaptations involving neural drive (number of active motor units and their rate coding), motor unit recruitment threshold, and their type^6,37^. SEMG, while alone without for example the assessment of voluntary activation though interpolation twitch technique or muscle fibers conduction velocity cannot distinguish the nature of the fatigue resistance, it can give insight into neuromuscular adaptation of long-term sport training^38,39^. Typical changes in SEMG related to the fatigue in submaximal contractions are increase in muscle activity (higher amplitude) and shifts toward the lower band of the power spectrum decrease in frequency^40^. The tennis match comprises of both submaximal and maximal efforts and it lasts from 2 to 5 h and it is characterized with intermittent play time of 20–30% match time^41^. It was previously shown that, quadriceps femoris muscles decrease their activity along the tennis match^2,42^, however without change in power spectrum^43^. Moreover, studies showed that tennis players can exhibit distinct level of fatigue and recovery in particular parts of quadriceps muscle^44^. While the changes in neuromuscular fatigue of lower limbs related to the tennis match is well investigated, this is the first time that the muscle activity of tennis players after MAnE is shown.

Regardless superior PkRTD in MVIC knee extension, TP did not exhibit specific activity of quadriceps femoris muscle in terms of amplitude or median frequency of power spectrum as agonist. However, during MVIC knee flexion, when quadriceps femoris muscle act as antagonist, the SEMG activity showed difference in comparison to the PAM, especially in rectus femoris muscle. Overall, CI of rectus femoris in TP was higher than in PAM and it also showed tendency to increase after the MAnE. Coactivation of antagonist muscles can hinder the level of produced torque, however in case of rapid force/torque development its role could be different, i.e. stabilizing and controlling the joint to quickly develop the output, especially in early time of RTD^45,46^.

To investigate further the neural aspects of neuromuscular performance, participants carried out 20% and 50% MVIC tasks. Typically, all investigated muscles in TP: rectus femoris, vastus medialis, vastus lateralis showed overall lower muscle activity during 50% MVIC of knee extension., which reflects the neuronal adjustments representing increasing neuromuscular efficiency that results from sport training^12,13^.

Considering knee flexion, the CI of rectus femoris in 20% MVIC increased after the MAnE in TP, which was associated with overall lower median frequency of power spectrum in TP. This suggest that TP, after the MAnE, may recruit more slow-type motor units or synchronize them more to maintain performance of fatigued hamstring muscles. Similar increase in CI of TP’s vastus medialis muscle in 20% MVIC was observed. On the other hand, in PAM, while the agonists muscle activity did not change after MAnE, they were overall characterized by higher CI of vastus lateralis in 50% MVIC task. These changes and differences in SEMG may be reflection of specific muscle tennis-related sport training adaptations regarding not only the coactivation of quadriceps femoris as antagonist but also particular parts of the muscle itself^47,48^. It must be pointed out that reported results in submaximal contractions i.e. 20% and 50% of MVIC, were evaluated in slow and precise contraction not as during MVIC for PkRTD evaluation. Therefore, presented submaximal contraction outcome cannot be directly related to the PkRTD performance, but rather show general neuromuscular coordination changes related to the fatigue after MAnE. However, as there were no differences in response to the MAnE in terms of PkTQ between groups, the observed changes in SEMG in TP, should be in some extend associated with ability to maintain high level of knee flexors explosive strength after the MAnE. Further investigations involving RTD analysis in submaximal contractions should be performed to verify if the outcome would be similar.

Overall, these results indicate that elite TP can exhibit specific neuromuscular adaptation that allows them to maintain the explosive muscle strength performance after MAnE in form of WAnTs.

### Biochemical response

Biochemically, the present study extends evidence on tennis-trained athletes by quantifying acute post-MAnE inflammatory and redox responses in elite players relative to physically active controls. The results indicate distinct post-exercise inflammatory profiles between professional TP and PAM, underscoring the role of long-term training in modulating immune responses. While a robust acute cytokine response was evident across participants, irrespective of group, IL-6 (75.0%) and IL-10 (76.9%) increased markedly immediately after MAnE, which aligns with previous findings indicating that intense exercise induces an acute inflammatory response^49^. In this context, IL-6 is considered an important mediator of exercise-induced muscle repair, metabolic regulation, and adaptation following intensive exercise^50^. Accordingly, the pronounced increase in IL-10, an anti-inflammatory cytokine, may represent a compensatory response counterbalancing the pro-inflammatory milieu associated with elevated IL-6^51^. In contrast, IL-15 decreased significantly immediately after MAnE (13.5%) without between-group differences; although meta-analytic evidence indicates that circulating IL-15 typically increases immediately post-exercise on average, the substantial heterogeneity of responses and the short half-life of IL-15 support the possibility of protocol- and sampling-dependent directional differences^52^. Against this background, TP exhibited lower IL-6 concentrations than PAM at baseline and a less pronounced post-exercise IL-6 response immediately and 3 h after MAnE. Although IL-6 is often considered a pro-inflammatory cytokine, exercise-induced IL-6 is also released from contracting skeletal muscle and acts as a myokine involved in metabolic regulation and anti-inflammatory signaling^51,53^. Therefore, the attenuated IL-6 response in TP, despite higher anaerobic performance, may reflect a lower relative inflammatory challenge in athletes chronically exposed to repeated high-intensity intermittent efforts^54,55^.

The concomitantly higher IL-10 concentrations in TP further support this interpretation. IL-10 is a key anti-inflammatory cytokine that suppresses pro-inflammatory mediator production and contributes to immunoregulatory balance^56^. Moreover, exercise-induced IL-6 has been shown to stimulate the appearance of anti-inflammatory mediators, including IL-10, which supports the interpretation of IL-10 as part of the compensatory anti-inflammatory response after strenuous exercise^54^. Thus, the combination of lower IL-6 and higher IL-10 immediately and 3 h after MAnE suggests a more favorable anti-inflammatory profile in TP than in PAM. This response may represent a long-term training-related adaptation^54,55^, although the systemic cytokine measurements used in the present study do not allow identification of the specific cellular or molecular mechanisms involved.

Beyond cytokine dynamics, indices of oxidative stress and plasma protein status were assessed to contextualize the redox response. The observed post-exercise decrease in TAC (47.5%) in TP suggests a transient reduction in the body’s antioxidant defenses, which could be attributed to the oxidative stress induced by high-intensity exercise which likely reflects an acute ROS challenge induced by the high-intensity bouts and the greater metabolic strain associated with higher anaerobic performance^10^. Moreover, the baseline levels of TAC were notably higher in TP compared to PAM, suggesting that regular training may enhance athletes’ antioxidant capacity. This finding is consistent with research by Gomez-Cabrera et al.^57^, Kochanowicz et al.^18^, who noted that trained individuals often exhibit superior antioxidant capabilities and differences in post-exercises induced inflammation compared to untrained populations. The sustained higher TAC levels in TP population 24 h after MAnE (34.7%) further support the conclusions that regular tennis training can bolster recovery and induces some biochemical adaptations. While acute exercise typically elevates inflammation markers due to membrane disruption, chronic training enhances mitochondrial biogenesis and antioxidant defenses, reducing oxidative stress and muscle damage^58,59^.

8-OHdG and ALB showed no significant main or interaction effects, indicating lower acute sensitivity to the presented MAnE than the robust alterations in IL-6, IL-10, IL-15 and TAC. Transient ROS production induced by prolonged exercises induces a protracted elevation in 8-OHdG as a product of DNA oxidation^60^. 8-OHdG serve as a danger-associated molecular pattern, triggering an immune response to promote tissue regeneration but mostly in long lasting inflammation^61,62^. It is important to note that changes in 8-OHdG may be directly associated with the increase of post-exercises inflammation and ROS production. In presented research the small, and non-significant changes of 8-OHdG likely reflect the brief duration of the WAnTs test—short high-intensity efforts^63,64^. This contrasts with our previous research where we used a prolonged shuttle-run protocol to induced muscle damage and 8-OHdG increased by 38% immediately post-exercise^65^. Similar like 8-OHdG, serum ALB showed no significant main or interactions effects. Therefore, total serum ALB concentration should not be interpreted as direct evidence of antioxidant defense adaptation.

The small post-exercise fluctuations observed in ALB may reflect transient hemoconcentration/hemodilution or plasma volume shifts rather than true changes in ALB mass. Moreover, redox modification of the Cys34 thiol may occur without altering ALB total concentration; together with the 17–19-day half-life of ALB, this explains the small, non-significant shifts after a MAnE^66–68^. Conversely, IL-6/IL-10 exhibit rapid myokine kinetics, and TAC reflects immediate adjustments in systemic antioxidant defenses to the acute ROS challenge^15,18,54^. These differences align with established literature on exercise-induced adaptations and provide insight into systemic biochemical responses to standardized maximal anaerobic stress in tennis-trained athletes.

### Limitations

Several limitations should be acknowledged. First, the study included only male participants; therefore, the present findings should not be directly generalized to female tennis players. This is relevant because sex-related differences may influence neuromuscular fatigability and exercise-induced inflammatory responses, including cytokine regulation. In women, these responses may also be affected by menstrual cycle phase^69–71^.

Second, performing MAnE requires full commitment from participants, and in non-training individuals who are not accustomed to maximal effort, the obtained results may not fully represent their anaerobic potential. To limit this possibility, each participant completed a familiarization session with the study protocol before the main testing procedures.

Another limitation is that MAnE was induced using a lower-body WAnT, whereas tennis performance relies on a coordinated whole-body kinetic chain. Thus, WAnT should be interpreted as a standardized maximal anaerobic fatigue stimulus rather than a tennis-specific movement task, and direct transfer to on-court performance should be made with caution.

Another methodological limitation is the lack of hamstring muscle activity evaluation. The study setup prioritized neuromuscular performance assessment in the form of RTD during knee extension and flexion, given its relevance to sport-specific and functional tasks^72,73^. While there was an intention to investigate the biceps femoris activity, besides the quadriceps femoris muscle, the experimental setup involving sitting position involving mechanical pressure on the electrodes was the cause of many SEMG signal artifacts in the measurements. Therefore, in the study biceps femoris SEMG activity is not presented, and study was focused on quadriceps femoris muscle as example of neuromuscular adaptations. Further studies investigating the long-term tennis training effects should include hamstrings muscle activity evaluation to supplement and expand the understanding of proposed neuromuscular changes.

The final limitation is that explosive strength was evaluated only in form of PkRTD. It is common to evaluate RTD in form of distinct time intervals or as time to achieve percentage torque level due to the fact that different factors influence early (< 100 ms) and late (> 100 ms) RTD^74^. However, as the PkRTD is one of the most sensitive indicator of RTD^6^ and with the intention of concisely presenting the various aspects of fatigue the presented approach seems reasonable.

Beyond these methodological limitations, future research could extend the present findings by including TP across different competitive levels. A comparison between high-ranked and lower-ranked national players would address a related but distinct research question and may help determine whether the observed neuromuscular and biochemical adaptations vary according to competitive standard.

## Conclusions

Elite tennis players demonstrated an integrated pattern of long-term adaptations characterized by better preservation of explosive torque production after MAnE, altered SEMG-derived neuromuscular responses, and a more favorable post-exercise inflammatory and redox-related profile compared with physically active men. These findings suggest that long-term high-performance tennis training may be associated with enhanced fatigue resistance and more efficient post-exercise regulation in response to standardized MAnE.

From a sport science perspective, the present findings provide insight into coordinated neuromuscular and biochemical responses to supramaximal anaerobic stress in elite male tennis players. The distinct inflammatory and redox-related response patterns observed in trained individuals also highlight the importance of considering training status when interpreting exercise-induced physiological changes. Such adaptations may enhance the capacity to regulate oxidative stress and inflammation, thereby supporting post-exercise recovery processes. Given the whole-body, technical, and intermittent nature of tennis match play, further studies should evaluate the relevance of these adaptations using protocols that more closely reflect on-court movement demands and should determine the cellular and molecular mechanisms underlying the observed responses.

## Data Availability

All data analyzed during this study are available from the main author of the reasonable request.
